# Multidrug Resistance *Salmonella* Genomic Island 1 in a *Morganella morganii* subsp. *morganii* Human Clinical Isolate from France

**DOI:** 10.1128/mSphere.00118-17

**Published:** 2017-04-19

**Authors:** Eliette Schultz, Olivier Barraud, Jean-Yves Madec, Marisa Haenni, Axel Cloeckaert, Marie-Cécile Ploy, Benoît Doublet

**Affiliations:** a ISP, INRA, Université Tours, UMR 1282, Nouzilly, France; b Unité Antibiorésistance et Virulence Bactériennes, Université Lyon—ANSES Site de Lyon, Lyon, France; c Université Limoges, INSERM, CHU Limoges, UMR 1092, Limoges, France; Escola Paulista de Medicina/Universidade Federal de São Paulo

**Keywords:** *Salmonella* genomic island 1, integrative mobilizable element, integrons, multidrug resistance

## Abstract

*Salmonella* genomic island 1 (SGI1) is a multidrug resistance integrative mobilizable element that harbors a great diversity of antimicrobial resistance gene clusters described in numerous *Salmonella enterica* serovars and also in *Proteus mirabilis*. A serious threat to public health was revealed in the recent description in *P. mirabilis* of a SGI1-derivative multidrug resistance island named PGI1 (*Proteus* genomic island 1) carrying extended-spectrum-β-lactamase (ESBL) and metallo-β-lactamase resistance genes, *bla*_VEB-6_ and *bla*_NDM-1_, respectively. Here, we report the first description of *Salmonella* genomic island 1 (SGI1) in a multidrug-resistant clinical *Morganella morganii* subsp. *morganii* strain isolated from a patient in France in 2013. Complete-genome sequencing of the strain revealed SGI1 variant SGI1-L carrying resistance genes *dfrA15*, *floR*, *tetA*(G), *bla*_PSE-1_ (now referred to as *bla*_CARB-2_), and *sul1*, conferring resistance to trimethoprim, phenicols, tetracyclines, amoxicillin, and sulfonamides, respectively. The SGI1-L variant was integrated into the usual chromosome-specific integration site at the 3′ end of the *trmE* gene. Beyond *Salmonella enterica* and *Proteus mirabilis*, the SGI1 integrative mobilizable element may thus also disseminate its multidrug resistance phenotype in another genus belonging to the *Proteae* tribe of the family *Enterobacteriaceae*.

**IMPORTANCE** Since its initial identification in epidemic multidrug-resistant *Salmonella enterica* serovar Typhimurium DT104 strains, several SGI1 variants, SGI1 lineages, and SGI1-related elements (SGI2, PGI1, and AGI1) have been described in many bacterial genera (*Salmonella*, *Proteus*, *Morganella*, *Vibrio*, *Shewanella*, etc.). They constitute a family of multidrug resistance site-specific integrative elements acquired by horizontal gene transfer, SGI1 being the best-characterized element. The horizontal transfer of SGI1/PGI1 elements into other genera is of public health concern, notably with regard to the spread of critically important resistance genes such as ESBL and carbapenemase genes. The identification of SGI1 in *Morganella morganii* raises the issue of (i) the potential for SGI1 to emerge in other human pathogens and (ii) its bacterial host range. Further surveillance and research are needed to understand the epidemiology, the spread, and the importance of the members of this SGI1 family of integrative elements in contributing to antibiotic resistance development.

## Abstract (precis)

Since its initial identification in epidemic multidrug-resistant *Salmonella enterica* serovar Typhimurium DT104 strains, several SGI1 variants, SGI1 lineages, and SGI1-related elements (SGI2, PGI1, and AGI1) have been described in many bacterial genera (*Salmonella*, *Proteus*, *Morganella*, *Vibrio*, *Shewanella*, etc.). They constitute a family of multidrug resistance site-specific integrative elements acquired by horizontal gene transfer, SGI1 being the best-characterized element. The horizontal transfer of SGI1/PGI1 elements into other genera is of public health concern, notably with regard to the spread of critically important resistance genes such as ESBL and carbapenemase genes. The identification of SGI1 in *Morganella morganii* raises the issue of (i) the potential for SGI1 to emerge in other human pathogens and (ii) its bacterial host range. Further surveillance and research are needed to understand the epidemiology, the spread, and the importance of the members of this SGI1 family of integrative elements in contributing to antibiotic resistance development.

## OBSERVATION

*Salmonella* genomic island 1 (SGI1) is a multidrug resistance (MDR) site-specific integrative mobilizable element (IME) initially described in *Salmonella* that integrates into the last 18 bp of the conserved chromosomal *trmE* gene (formerly *thdF*) (1, 2). Among the most prevalent incompatibility groups of plasmids, only the conjugative plasmids of the IncA/C family have been shown to specifically mobilize SGI1 in *trans* (3). Recently, the major IncA/C-encoded transcriptional activator complex, AcaCD, was shown to trigger SGI1 excision and in *trans* conjugative mobilization (4). SGI1 contains a complex class 1 integron, named In104 in accordance with its initial host strain (1, 5, 6). Since the identification of SGI1 in *Salmonella enterica* serovar Typhimurium DT104, the high genetic plasticity of its MDR region, highlighted by the diversity of the class 1 integron resistance gene cassettes and by the presence of recombination and insertion sequence (IS) element-mediated rearrangements, has led to the description of more than 30 different MDR regions of SGI1 in many *S. enterica* serovars (7–14). In addition, genetic variations are observed also in the backbone of the island, i.e., IS-mediated insertion/deletion, single nucleotide polymorphism (SNP), and transpositional insertion of the complex class 1 integron structure at another position (8, 10, 12, 14).

In 2006, SGI1 was identified in a clinical *Proteus mirabilis* strain from a diabetic patient from Palestine (15). Since then, the number of reported cases of SGI1 variants of this bacterial species in isolates from humans, food-producing animals, foodstuffs, and companion animals in China and France has been increasing (16–23). Recently, a novel SGI1 derivative MDR genomic island named *Proteus* genomic island 1 (PGI1) has been described in human and animal* P. mirabilis* strains in France (20, 21, 24, 25). PGI1 showed gene synteny similar to that of SGI1 and was also found integrated into the last 18 bp of the conserved chromosomal *trmE* gene. The recent emergence of *P. mirabilis* strains carrying SGI1 or PGI1 islands with extended-spectrum-β-lactamase and/or metallo-β-lactamase resistance genes, *bla*_VEB-6_ and *bla*_NDM-1_, respectively, representing the first description of the latter gene in an MDR genomic island, is a serious threat to public health (25). In this study, we analyzed the first SGI1-positive *Morganella morganii* subsp. *morganii* (here *M. morganii*) strain isolated from a human case in France.

A hepatitis C virus (HCV)- and human immunodeficiency virus (HIV)-positive 52-year-old man was hospitalized with high blood pressure and cirrhosis complicated by a hepatocellular carcinoma in January 2013 at the Limoges University Hospital center in France. The patient mentioned having had prostatitis in December 2012. MDR *M. morganii* strain LIM90 was isolated from urine sample during his stay at the hospital in January 2013. The strain was screened for antibiotic susceptibility by the disc diffusion method according to the guidelines of the EUCAST committee (26). Besides intrinsic resistance to several β-lactam antibiotics (amoxicillin ± clavulanic acid, cephalotin, cefuroxim), tetracycline, nitrofurantoin, fosfomycin, and colistin, *M. morganii* strain LIM90 was resistant to chloramphenicol, florfenicol, streptomycin, spectinomycin, sulfonamides, trimethoprim, and ticarcillin, which suggested the possible presence of SGI1. The result of PCR performed using primers corresponding to the integrase genes of SGI1 and related islands (FwintSGI1HR [5′-ATGTTGCGTCAGGCYGAGGC-3′] and RvintSGI1HR [5′-GAGTGYCCAAGAAGSCGAGAG-3′]) was positive, suggesting the presence of a SGI1-related island in *M. morganii* strain LIM90.

To identify the SGI1-related island, its chromosomal location, and its resistance gene content, the whole genome of LIM90 was sequenced using an IonProton system (99-fold average read depth). The reads were assembled using MIRA software. The ResFinder and PlasmidFinder tools available at the Center for Genomic Epidemiology were used for identification of acquired resistance genes and plasmid detection, respectively (27). The complete sequence of SGI1 was assembled using the relevant contigs detected by BLAST searches, PCR gap closure, and PCR product sequencing. The sequence of SGI1 was annotated using the Microbial Genome Annotation and Analysis Platform MicroScope (Genoscope, France) and deposited in the European Nucleotide Archive (ENA) under accession number LT630458 (28).

**FIG 1:**
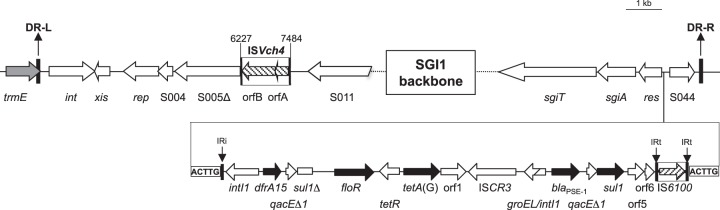
Schematic view of SGI1-L and its specific features encountered in *Morganella morganii* strain LIM90. The gray arrow corresponds to chromosomal gene *trmE* into which SGI1 is integrated into the last 18 bp. DR-L and DR-R are the 18-bp left and right direct repeats, respectively, bracketing SGI1. The insertion points of complex class 1 integron InSGI1-L between the *res* gene and ORF S044 of the SGI1 backbone and the 5-bp target site duplication are indicated. IRi and IRt are 25-bp imperfect inverted repeats defining the left and right end of the complex class 1 integron. Black arrows correspond to SGI1 antibiotic resistance genes. IS elements are indicated by boxes containing black hatched arrows representing the transposase genes. Base pair coordinates are from the complete SGI1-L sequence of *M. morganii* strain LIM90 (ENA accession no. LT630458).

*M. morganii* strain LIM90 harbored the SGI1-L variant shown in Fig. 1. SGI1-L-related islands have been previously described in *S. enterica* serovars and *P. mirabilis* strains but were never fully sequenced (10, 14). This SGI1-L variant carried the *dfrA15* and *bla*_CARB-2_ (previously named *bla*_PSE-1_) resistance gene cassettes inserted at the two SGI1 integron *attI* recombination sites, conferring resistance to trimethoprim and ticarcillin, respectively (Fig. 1). The *floR* gene, which confers resistance to chloramphenicol and florfenicol, and tetracycline resistance genes *tetR*(G) and *tetA*(G) were found to be flanked by these two integron structures (Fig. 1). The presence of IS*Vch*4 (also called IS*1359*) at position 6227 to position 7484 of the SGI1 backbone sequence (ENA accession number LT630458) was found as previously described in a few SGI1 variants (SGI1-H, SGI1-K, and SGI1-L derivatives) in *S. enterica* serovars and *P. mirabilis*. An adjacent 2,779-bp deletion removed the region extending from within open reading frame (ORF) S005 to within ORF S009 (Fig. 1). The absence of target site duplication created upon IS*Vch4* insertion suggested that additional recombinational events may have occurred after the transposition of this IS*Vch4* copy, i.e., replicative transposition of IS*Vch4* into ORF S009 and subsequent recombination between the two copies of IS*Vch4* creating the 2,779-bp deletion.

The backbone of SGI1-L (24,093 bp, excluding mobile elements like IS*Vch4* and the complex class 1 integron structure) was identical to that of SGI1 variants harboring the insertion/deletion of IS*Vch4* and previously described in *P. mirabilis* and *S*. Kentucky ST198 (Table 1) (13, 18). In addition to the IS*Vch4* insertion/deletion, the backbone of this group of SGI1 variants showed two single-base differences from other fully sequenced variants (29). One SNP was identified compared with IS*Vch4*-negative SGI1 of epidemic *S*. Typhimurium DT104 strains, resulting in an amino acid (AA) change in toxin SgiT (ORF S025) of the *sgiAT* toxin-antitoxin addiction system (Table 1) (28, 29). The putative role of this AA change in the SgiAT toxin-antitoxin system and in the stability of SGI1 remains to be determined. Concomitantly with the first SNP position described above, a second one was found in antitoxin SgiA (ORF S026, no AA change) of IS*Vch4*-negative variants described in other *S. enterica* serovars and *P. mirabilis* strains (Table 1) (29, 30). Interestingly, the original SGI1 sequence of *S*. Typhimurium (GenBank accession number AF261825), the recently released SGI1-F sequence of *S*. Cerro (GenBank accession no. KU847976), and the SGI2 sequence of *S*. Emek showed 7, 7, and 95 different base pair substitutions relative to the IS*Vch4*-positive SGI1 backbone, respectively (Table 1). All these SGI1 backbone characteristics suggest different lineages that evolve and horizontally spread among *Enterobacteriaceae*.

**TABLE 1:** Characteristics of complete SGI1 sequences and backbone SNP analysis

Host strain	SGI1 variant	IS*Vch4* indel	SNP position^a^		GenBank accession no.
			22001 (in *sgiT*)	24286 (in *sgiA*)	
*M. morganii*	SGI1-L	+	C	G	LT630458
*S*. Kentucky	SGI1-K	+	C	G	AY463797
*P. mirabilis*	SGI1-PmMAT	+^b^	C	G	JX089583
*P. mirabilis*	SGI1-PmABB	+	C	G	KP313760
*P. mirabilis*	SGI1-PmGUE	+	C	G	JX121641
*P. mirabilis*	SGI1-PmVER	+	C	G	JX121640
*P. mirabilis*	SGI1-PmSCO	+	C	G	JX121639
*P. mirabilis*	SGI1-PmABB	+	C	G	JX121638
*S*. Typhimurium	SGI1	−	A	G	KU499918
*S*. Typhimurium	SGI1	−	A	G	CP014979
*S*. Typhimurium	SGI1	−	A	G	CP014975
*S*. Typhimurium	SGI1	−	A	G	CP014969
*S*. Typhimurium	SGI1	−	A	G	CP014967
*S*. Typhimurium	SGI1	−	A	G	CP012985
*S*. Typhimurium	SGI1	−	A	G	CP014358
*S*. Typhimurium	SGI1	−	A	G	CP007581
*S*. Typhimurium	SGI1	−	A	G	HF937208
*S*. Infantis	SGI1-D	−	A	G	KU854986
*S*. Derby	SGI1-I	−	A	T	KU563154
*S*. Rissen	SGI1-I	−	A	T	KM234279
*P. mirabilis*	SGI1-B0616	−	A	T	KU987432
*P. mirabilis*	SGI1-O	−	A	T	KU987431
*P. mirabilis*	SGI1-B	−	A	T	KU987430
*P. mirabilis*	SGI1-Z	−	A	T	KP662516
*P. mirabilis*	SGI1-X	−	A	T	KJ186154
*P. mirabilis*	SGI1	−	A	T	KJ186153
*P. mirabilis*	SGI1-I	−	A	T	KJ186152
*P. mirabilis*	SGI1-W	−	A	T	KJ186151
*P. mirabilis*	SGI1-O	−	A	T	KJ186150
*P. mirabilis*	SGI1-Y	−	A	T	KJ186149
*P. mirabilis*	SGI1-PmBRI	−	A	T	JX089582
*P. mirabilis*	SGI1-PmCAU	−	A	T	JX089581
*P. mirabilis*	SGI1-B2	−	A	T	KP116299
*S*. Typhimurium	SGI1^c^	−	A	G	AF261825
*S*. Cerro	SGI1-F^d^	−	A	T	KU847976
*S*. Emek	SGI2^e^	−	A	G	AY963803

a SNP positions in the ORF of the TA system *sgiAT* are given according to ENA accession no. LT630458.

b The SGI1-PmMAT variant harbored the deletion created by IS*Vch4* and extending from within ORF S005 to within ORF S009 but without the presence of IS*Vch4* (18).

c The original SGI1 sequence (AF261825) showed 6 other specific SNP positions.

d The SGI1-F variant showed 5 other specific SNP positions.

e The SGI2 variant harbored 93 additional SNP positions and the transpositional insertion of the complex class 1 integron structure at a position different that in from all other variants (7, 8, 11, 14).

The whole-genome sequence analysis of *M. morganii* LIM90 revealed the presence of other antibiotic resistance determinants in the chromosome: (i) chromosomal AmpC β-lactamase gene *bla*_DHA-17_, conferring resistance to amoxicillin ± clavulanic acid, cephalotin, and cefuroxim (further confirmed by the phenotypic cloxacillin disk diffusion test; data not shown), and (ii) a class 2 integron carrying the *sat2* and *aadA1* gene cassettes, conferring resistance to streptothricin and streptomycin/spectinomycin, respectively. No plasmid of *Enterobacteriaceae* was detected by the PlasmidFinder tool, indicating that *M. morganii* LIM90 did not carry a conjugative IncA/C plasmid known to specifically mobilize SGI1 (2, 3). This observation is in accordance with the recently described incompatibility between SGI1 and members of the IncA/C plasmid family (30).

The identification of SGI1 in a *M. morganii* clinical isolate is of great interest, as the spread of this multidrug-resistant genomic island, especially in a naturally β-lactam-resistant species such as *M. morganii*, is a nonnegligible threat to public health. The important role of the horizontal transfer of SGI1 is crucial in the dissemination of multidrug resistance and may increase through pathogenic or nonpathogenic bacterial genera as well.

### Accession number(s).

The sequence of SGI1 was deposited in the European Nucleotide Archive (ENA) under accession number LT630458.
